# Porcine hemagglutinating encephalomyelitis virus VW572 (not Gent/PS412 and Labadie) uses the CD81 receptor and MVB-derived exosomal pathway for efficient entry and spread in neuronal cells

**DOI:** 10.1128/jvi.01171-25

**Published:** 2025-10-08

**Authors:** W. Zaib, C. Kaviani, X. Kang, Y. Gao, F. Vanden Broucke, W. Van den Broeck, S. Coppens, S. Theuns, H. Nauwynck, K. Laval

**Affiliations:** 1 Laboratory of Virology, Department of Translational Physiology, Infectiology and Public Health, Faculty of Veterinary Medicine, Ghent University 26656, Merelbeke, Belgium; 2 UGent TEM Core Facility, Department of Morphology, Imaging, Orthopedics, Rehabilitation and Nutrition, Faculty of Veterinary Medicine, Ghent University 26656, Merelbeke, Belgium; 3 PathoSense BV, Gent, Belgium; Loyola University Chicago - Health Sciences Campus, Maywood, Illinois, USA

**Keywords:** PHEV, neuropathogenesis, CD81 receptor, MVB-derived exosomal pathway

## Abstract

Porcine hemagglutinating encephalomyelitis virus (PHEV) is considered a neurotropic coronavirus that invades the peripheral (PNS) and central (CNS) nervous system of the pig and causes acute encephalomyelitis, also known as “vomiting and wasting disease.” Recently, PHEV has been proposed as a potential surrogate virus model to further elucidate the neuropathogenesis of other betacoronaviruses. In this study, we compared key steps in the replication cycle of three distinct PHEV isolates (VW572, Gent/PS412, and Labadie) in mouse neuronal (N2a) cells. We found that PHEV-VW572 replicates more efficiently in these cells compared to the other two isolates. Interestingly, PHEV-VW572 showed high intracellular virus titers without efficient extracellular release. Further investigation revealed that PHEV-VW572, but not PHEV-Gent/PS412, mainly uses multivesicular body (MVB)-derived exosomes for viral egress. Transmission electron microscopy confirmed the presence of complete PHEV-VW572 virions within intracellular vesicles and the release of fused PHEV-exosome structures near the plasma membrane. Finally, we showed that PHEV binding is restricted for all isolates. Still, we demonstrated that only PHEV-VW572 entry into cells is mediated by the tetraspanin CD81 receptor. Overall, these results suggest that PHEV-VW572 uses the MVB-derived exosomal pathway as a strategy to promote efficient infection and overcome the early restriction in neuronal cells. In addition, these findings highlight isolate-specific differences in PHEV neurotropism.

**IMPORTANCE:**

The neuropathogenesis of betacoronaviruses remains largely unclear despite the global impact of the severe acute respiratory syndrome coronavirus 2 (SARS-CoV-2) pandemic. While these viruses are primarily known for their respiratory effects, mounting evidence suggests they can also cause significant neurological complications, ranging from mild symptoms such as headaches to severe outcomes, such as encephalitis and neurological diseases. The exact mechanisms by which coronaviruses affect the nervous system are still not fully understood, which hampers the development of adequate treatments and prevention strategies for these neurological disorders. In this study, we used the porcine hemagglutinating encephalomyelitis virus (PHEV) as a surrogate model for SARS-CoV-2 to further unravel the neuropathogenesis of betacoronaviruses.

## INTRODUCTION

Coronaviruses (CoVs) are common pathogens of humans and animals. While most cause mild to severe respiratory infections, CoVs are also known to exhibit neurotropic and neuroinvasive capabilities in several of their hosts, including rodents, swine, and humans (1–3). Since the coronavirus disease (COVID-19) pandemic, there is a growing interest in the pathogenesis of betacoronaviruses and their impact on society. Specifically, numerous COVID-19 cases associated with neurological manifestations have been reported over the years worldwide (4). Still, the exact role of the virus in this type of complication is not known. Therefore, a better understanding of the neuropathogenic potential of betacoronaviruses is urgently needed. So far, the mouse hepatitis virus has been used as an experimental platform to study the pathobiology of respiratory and neurological manifestations, similar to those of human-CoVs (5, 6). Under-studied, but equally relevant, the swine betacoronavirus, porcine hemagglutinating encephalomyelitis virus (PHEV), has recently been proposed as a surrogate model to study severe acute respiratory syndrome coronavirus 2 (SARS-CoV-2) infection (7). Like SARS-CoV-2, PHEV can similarly invade the peripheral nervous system (PNS) and spread to the central nervous system (CNS) in mice via the olfactory pathway, therefore causing neuroinflammation and neurological symptoms, suggesting that these two viruses may share common viral mechanisms for neuropathologies in their hosts.

PHEV is a pathogen of veterinary importance, causing acute encephalomyelitis, also known as “vomiting and wasting disease” in pigs (8). The virus circulates subclinically at a high prevalence in most swine herds worldwide. The virus is transmitted primarily through close contact and respiratory droplets and enters via the oral or nasal route. The incubation period is about 4 to 6 days. It can infect naïve pigs of any age, but clinical signs are age-dependent. Neonatal pigs that are born to naïve sows and become infected reach a mortality rate of 100%. The clinical signs include anorexia accompanied or followed within a few hours by vomiting. At 1 day post-infection (dpi), the nasal mucosa, tonsils, and lungs serve as primary replication sites. Alternatively, the virus can also replicate in the epithelial cells of jejunal villi after oral inoculation. From 2 to 3 dpi, PHEV spreads to the PNS. The virus progresses via the peripheral nerves innervating the primary replication site. Several pathways for viral dissemination can be used. The first viral spread pathway is from the nasal mucosa and tonsils to the trigeminal ganglion. A second pathway is from the lungs to the vagal nerve. A third pathway is from the small intestine to the solar ganglion. At 4 dpi, PHEV finally reaches the CNS (mainly pons and medulla). Viral replication and inflammation in the brain lead to death (9). To date, there is no vaccine available (10).

Here, we investigated the neuropathogenesis of PHEV by comparing the replication kinetics of three distinct PHEV isolates in mouse neuroblastoma (N2a) cells. We aimed to identify key similarities and/or differences in the replication cycle between isolates that may influence their neurotropic and neuroinvasive potential.

## RESULTS

### PHEV-VW572 infects N2a cells, but without efficient release of extracellular virus particles

**Fig 1:**
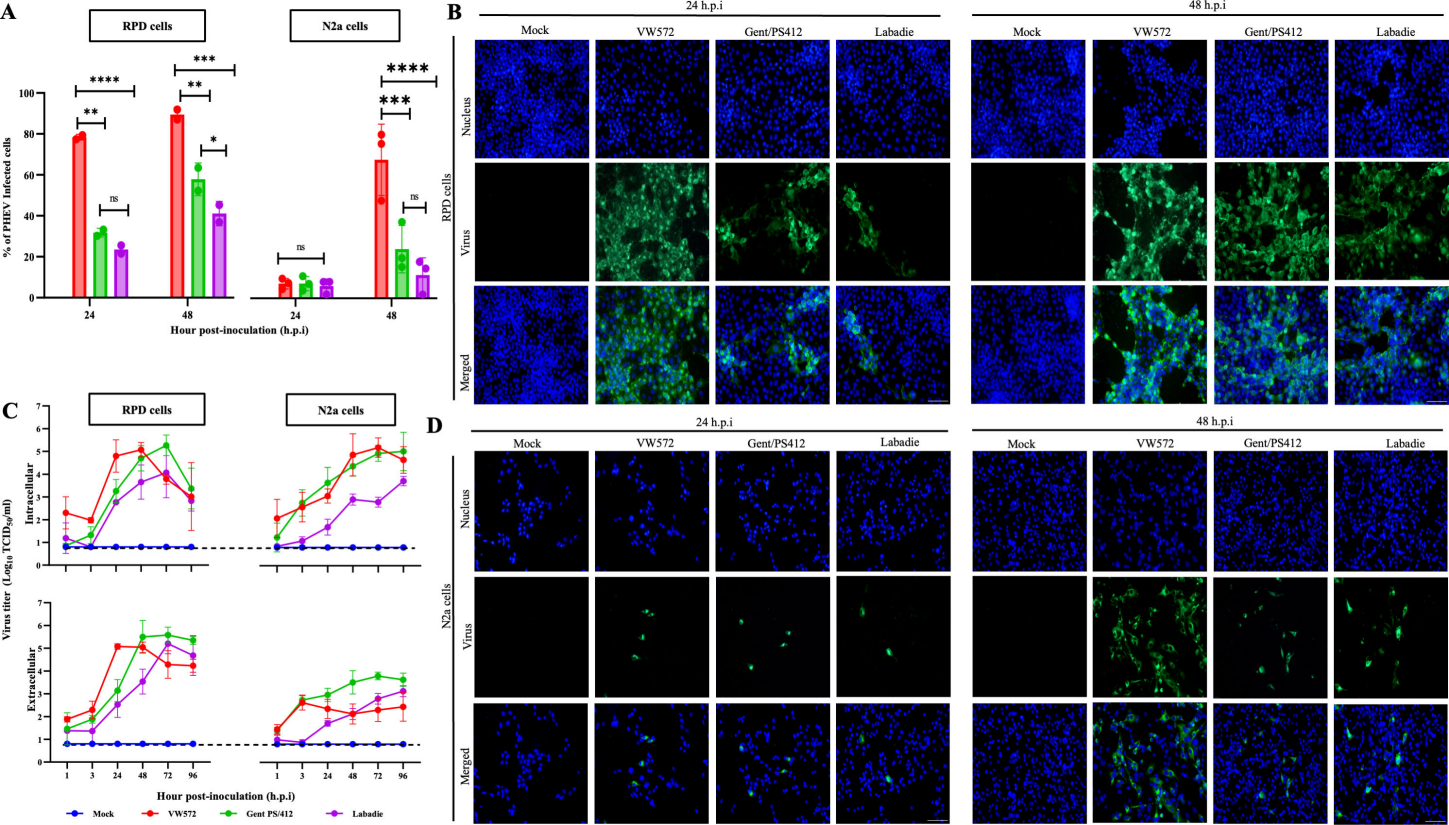
Comparative replication kinetics of PHEV isolates in RPD and N2a cells. (**A**) Kinetics of PHEV protein expression in RPD and N2a cells. Cells were (mock) inoculated at an MOI of 1 with either PHEV VW5.72, Gent/PS412, or Labadie. The percentage of PHEV-infected cells was quantified at 24 and 48 hpi by IF staining. Fixed cells were stained for PHEV S protein (green), and nuclei were counterstained with Hoechst 33342 (blue). Representative IF images of PHEV-infected RDP (**B**) and N2a (**D**) cells at 24 and 48 hpi. Scale bar represents 100 µm. (**C**) Kinetics of intracellular and extracellular virus titers in RPD and N2a cells. Virus titers are expressed as log_10_ TCID_50_/mL. Error bars indicate standard deviation (SD) and ns, not significant; *, *P* < 0.05; **, *P* < 0.01; ***, *P* < 0.001; ****, *P* < 0.0001.

We first characterized the replication kinetics of three PHEV isolates in N2a cells to determine their neuroinvasive potential compared to fully susceptible porcine kidney cells (RPD). As shown in Fig. 1A, the percentage of viral-infected RPD cells was significantly higher (approx 70%) at 24 hours post-inoculation (hpi) for PHEV-VW572 than for PHEV-Gent/PS412 and -Labadie isolates (approx 30%). At 48 hpi, the percentage of infected RPD cells reached >80% for PHEV-VW572 and approximately 60% and 40% for both PHEV-Gent/PS412 and Labadie isolates, respectively. In contrast, the percentage of PHEV-positive N2a cells remained low at 24 hpi and was comparable (approx 10%) between isolates. While the percentage of infected N2a cells increased to approximately 70% for the PHEV-VW572 isolate at 48 hpi, it did not significantly increase (approx 10%–20%) for PHEV-Gent/PS412 and Labadie isolates. At 72 hpi, most RPD cells inoculated with PHEV-VW572 detached due to infection. It was therefore not possible to perform counting. Still, the percentage of Gent/PS412 and Labadie infected N2a cells remained similar between 48 and 72 hpi (Fig. S1). A rate-limiting step of infection was observed in N2a cells infected with PHEV-Gent/PS412 and Labadie at a higher multiplicity of infection (MOI), but not for the PHEV-VW572 isolate (Fig. S2). Representative immunofluorescence (IF) pictures of the PHEV infection are depicted in Fig. 1B and D. No signal was detected in the mock condition. The PHEV S protein was expressed in the cytoplasm of RPD and N2a cells, as single infected cells and syncytia.

**Fig 2:**
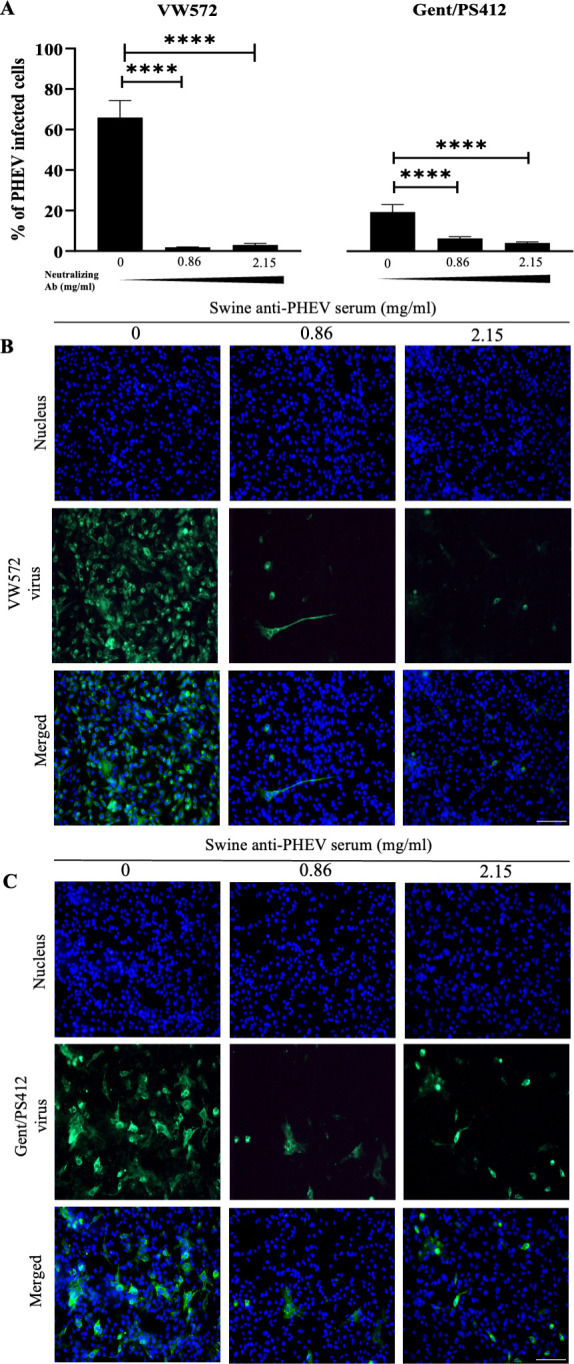
Neutralizing anti-PHEV antibodies inhibit PHEV spread in N2a cells. (**A**) Percentage of PHEV-infected cells in the presence or absence of neutralizing antibodies in culture medium. Cells were inoculated at an MOI of 1 with either PHEV VW572 or Gent/PS412 isolates for 1 h. After washing, cells were further incubated for 48 hpi in neuronal medium containing swine anti-PHEV serum at a concentration of 0, 0.86, or 2.15 mg/mL. Infection rates were quantified by IF staining. Error bars indicate standard deviation (SD) and ****, *P* < 0.0001. (**B** and **C**) Representative IF pictures of PHEV-infected cells following antibody treatment. Cells were stained for PHEV S protein (green), and nuclei were counterstained with Hoechst 33342 (blue). Scale bars represent 100 µm.

We also quantified the extracellular and intracellular PHEV titers in both inoculated cell types to confirm viral infectivity. As shown in Fig. 1C, an increase of intra- and extracellular PHEV titers in RPD cells was detected at 24 hpi for all isolates. Maximal titers were observed between 48 and 72 hpi. A decrease in PHEV intracellular titer starting from 72 hpi was noticed as most cells had been infected by that time. In N2a cells, newly replicated virus was detected for both PHEV-VW572 and Gent/PS412 isolates starting from 3 hpi. Intracellular viral titers increased over the course of the infection for all three isolates and reached comparable levels to those observed in RPD cells. In contrast, lower extracellular virus titers were detected in N2a cells than in RPD cells. While the PHEV extracellular titer increased from 24 to 72 hpi for both Gent/PS412 and Labadie, no significant increase was found for PHEV-VW572 in the neuronal cells throughout the course of the infection (<10^3^ 50% tissue culture effective dose [TCID_50_]/mL). Furthermore, quantitative PCR measurements of PHEV-VW572 N genomic RNA in cell lysates and supernatants confirmed viral titration results. Intracellular viral loads were significantly higher than extracellular ones in N2a cells at 48 and 72 hpi (Fig. S3).

Overall, we concluded that all three PHEV isolates replicated more efficiently in RPD than in N2a cells. While comparable intracellular PHEV titers were reached in both cell types at the late stage of infection, a significantly lower extracellular virus titer was observed in N2a compared to RPD cells throughout the course of the infection. Interestingly, a high intracellular titer with no increase of extracellular virus was observed in N2a cells inoculated with the VW572 isolate. These findings suggested that new progeny virions accumulate inside the cells with no efficient egress.

### Both PHEV-VW572 and PHEV-Gent/PS412 isolates do not spread cell-to-cell in N2a cells

Based on the above findings, we hypothesized that PHEV-VW572 spreads cell-to-cell in neuronal cells and does not release extracellular virus particles as part of an immune evasive strategy. Spike-induced cell-to-cell fusion is known to be important for efficient cell-to-cell spread of betacoronaviruses (11). To test this hypothesis, we compared the ability of PHEV-VW572 and PHEV-Gent/PS412 isolates to spread from cell to cell in the presence of PHEV-neutralizing antibodies. As PHEV-Gent/PS412 and PHEV-Labadie isolates showed similar viral replication kinetics, we only performed these experiments using PHEV-Gent/PS412 for direct comparison with the PHEV-VW572 isolate. As shown in Fig. 2A, treatment with viral neutralizing antibodies significantly inhibited PHEV spread in N2a cells in a dose-dependent manner. The percentage of PHEV-VW572-infected cells decreased significantly from approximately 70% (control) to approximately 3.5% (with treatment). A similar downward trend was observed for the Gent/PS412 isolate, where the percentage of infected cells went from approximately 20% to 3%. Representative IF pictures are provided in Fig. 2B and C. These findings demonstrate that PHEV does not spread cell-to-cell in N2a cells, independently of the viral isolate used. Therefore, these results do not support our hypothesis that PHEV-VW572 virions accumulate intracellularly and mainly spread cell-to-cell to evade antibody detection.

### PHEV-VW572, but not PHEV-Gent/PS412, uses MVB-derived exosomal pathway for egress in N2a cells

**Fig 3:**
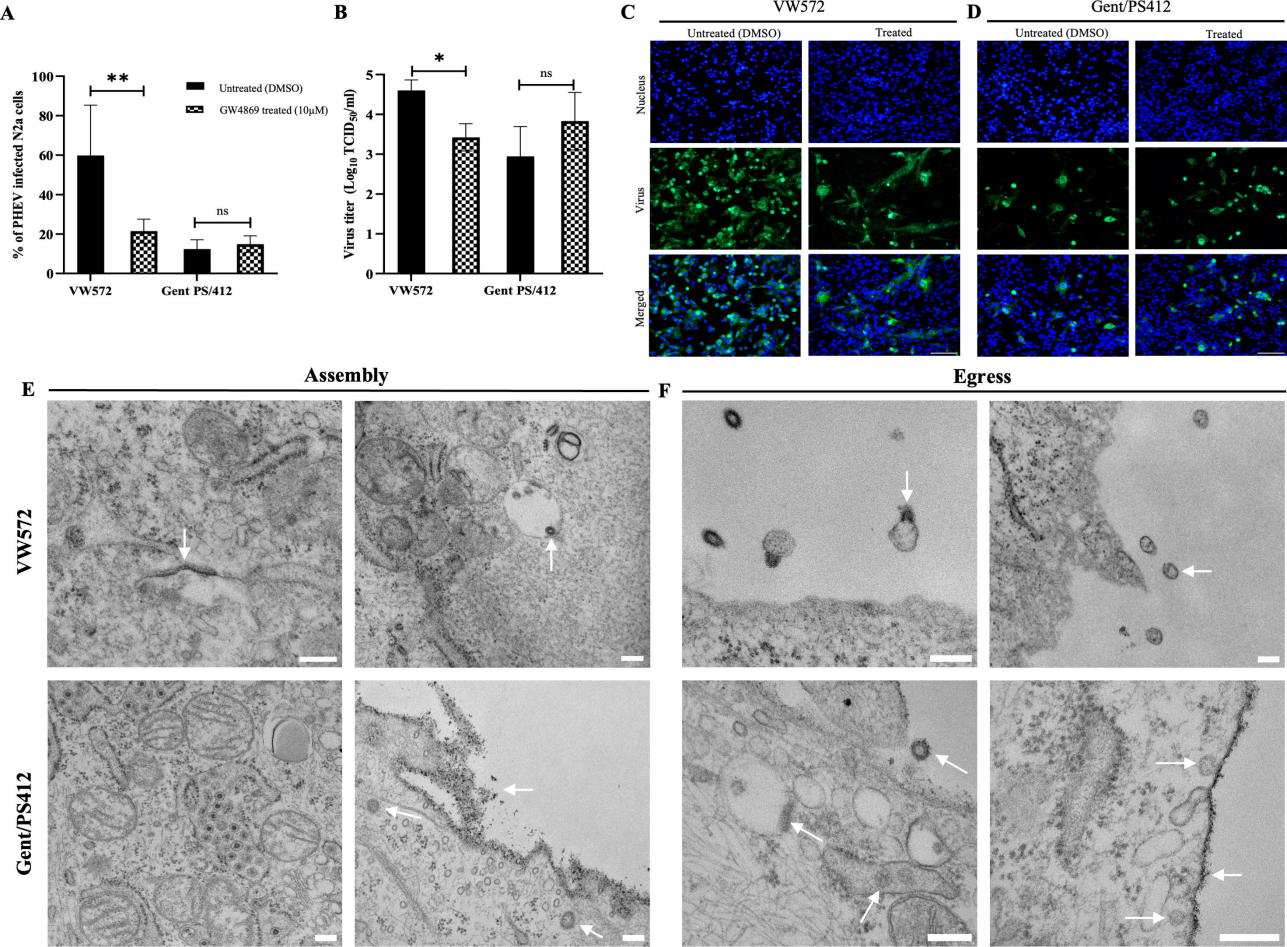
Comparative characterization of PHEV assembly and egress in N2a cells between viral isolates. (**A**) Percentage of PHEV-infected N2a cells and (**B**) quantification of PHEV titers (log_10_ TCID_50_/mL) following treatment with GW4869 (10 µM) or control (DMSO) at 48 hpi. Error bars indicate standard deviation (SD) and ns, not significant; *, *P* < 0.05; **, *P* < 0.01. (**C** and **D**) Representative IF images of PHEV-VW572 and -Gent/PS412-infected N2a cells upon GW4869 treatment. Cells were stained for PHEV S protein (green), and nuclei were counterstained with Hoechst 33342 (blue). Scale bars represent 100 µm. (**E** and **F**) Transmission electron microscopy (TEM) pictures of PHEV assembly and egress in N2a cells at 48 hpi. White arrows show signs of assembly or indicate the presence of virus particles. Scale bars represent 200 nm.

Betacoronaviruses are known to exploit either the lysosomal pathway or secretory vesicles for release into the extracellular environment (12–14). Recently, it was shown that PHEV can use the encapsulation of multiple viral components and host factors within multivesicular bodies (MVB) for efficient cell-to-cell communication (11). Still, it is not known whether this strategy is shared among all PHEV isolates or whether specific PHEV isolates use it only for efficient spread in neurons. Hence, we next aimed to examine whether PHEV-VW572 and Gent/PS412 isolates use the MVB-derived exosome pathway for viral egress in neuronal cells or not. Prior to viral inoculation, cells were pre-treated with GW4869, a specific inhibitor of neutral sphingomyelinase (nSMase), essential for exosome biogenesis and release. As shown in Fig. 3A, treatment of cells with 10 µM GW4869 significantly reduced the percentage of PHEV-infected cells from 75% to 30% at 48 hpi for the VW572 isolate. This coincided with a significant decrease in virus titer from 10^4.6^ to 10^3.5^ TCID_50_/mL (Fig. 3B). In contrast, GW4869 treatment did not affect PHEV infection with the Gent/PS412 isolate. Comparative IF pictures of treated versus untreated infected cells are provided in Fig. 3C and D. Additionally, cells treated with GW4869 after the 1 h viral inoculation showed a similar reduction in the percentage of infection for PHEV-VW572, demonstrating that the inhibitor did not affect the initial entry of viral particles to cells upon pre-treatment (Fig. S4). These findings indicate that PHEV-VW572, but not Gent/PS412 isolate, uses the MVB-derived exosomes for viral exit in neuronal cells.

**Fig 4:**
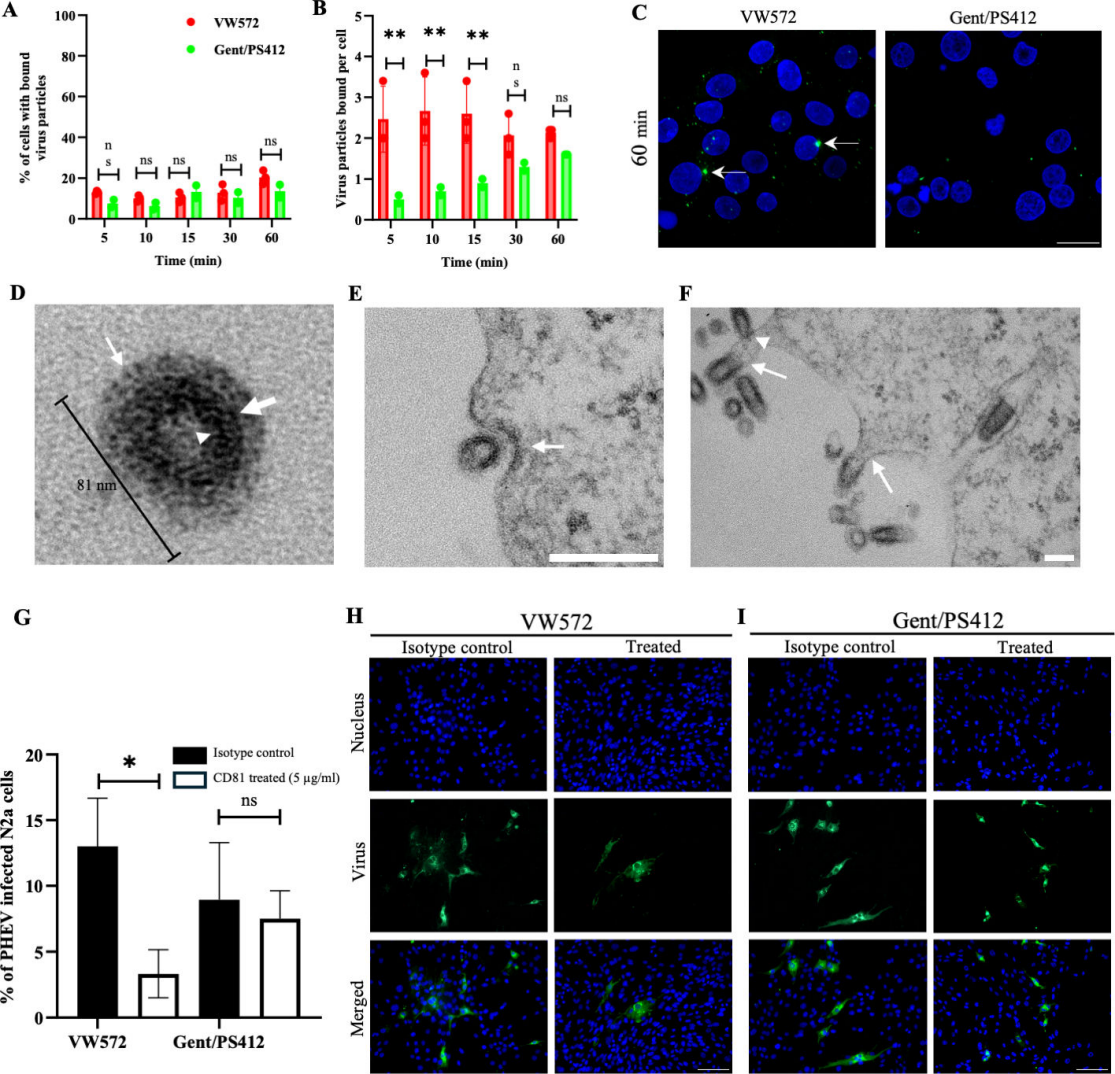
Characterization of PHEV entry into N2a cells between viral isolates. (**A**) Percentage of cells with bound PHEV particles at different times post-binding. Cells were inoculated at an MOI of 1 on ice with either PHEV VW572 or Gent/PS412 isolates. (**B**) Number of virus particles bound per cell at different time points. Error bars indicate standard deviation (SD) and ns, not significant; **, *P* < 0.01. (**C**) Representative IF images showing PHEV binding to cells at 60 min. The virus is labeled with Dio (green), and nuclei are stained with Hoechst 33342 (blue). Arrows show viral particle aggregates. Scale bar represents 10 µm. (**D**) Representative TEM of PHEV virion. Arrowhead represents helical nucleocapsid; thick arrow represents the envelope, and thin arrow represents club-shaped surface spike proteins. (**E** and **F**) Representative TEM pictures for PHEV entry by clathrin-mediated endocytosis (CME) or by direct fusion at 48 hpi. A thin arrow represents (CME) or fusion, and the arrowhead shows the start of entry with thickening of the membrane. Scale bars represent 200 nm. (**G**) Percentage of PHEV-infected cells at 24 hpi following treatment with anti-CD81 antibody (5 µg/mL) or isotype control. Error bars indicate standard deviation (SD) and ns, not significant; *, *P* < 0.05. (**H** and **I**) Representative IF pictures of PHEV-VW572 and -Gent/PS412-infected cells upon treatment. Cells were stained for PHEV S protein (green), and nuclei were counterstained with Hoechst 33342 (blue). Scale bars represent 100 µm.

Using transmission electron microscopy (TEM), we further investigated and compared the viral assembly and egress steps between both isolates. PHEV-VW572 was found to assemble in the ER-Golgi intermediate compartment (ERGIC) with the apposition of nucleocapsids along membranes of the budding compartment as particles developed and budded (Fig. 3E, upper panel). In addition, we observed the presence of complete virions within intracellular vesicles. PHEV-Gent/PS412 assembly also occurred in the ERGIC, but in addition to vesicles containing virions, some naked virus particles were seen, as well in the cytoplasm (Fig. 3E, lower panel). At the egress level, no clear signs of viral exit were detected for PHEV-VW572. No exocytosis, budding of virions at the plasma membrane, nor extracellular vesicles (EVs) containing virions were seen. Instead, we mainly observed fused virus-EV structures in the vicinity of the plasma membrane (Fig. 3F, upper panel). In contrast, clear viral exit sites and spike formation were seen at the plasma membrane of PHEV-Gent/PS412 infected cells (Fig. 3F, lower panel). Furthermore, the plasma membrane integrity was assessed in cells upon PHEV infection by TEM. While cells infected with PHEV-VW572 showed an intact plasma membrane, we found clear signs of plasma membrane disruption and compromised membrane continuity (indicative of cell lysis) in PHEV-Gent/PS412 infected cells (Fig. S5A). No significant change in the permeability of the plasma membrane of N2a cells was detected during viral infection following trypan blue and propidium iodide exclusion staining (Fig. S5B). Overall, these results supported the concept that PHEV-VW572, but not PHEV-Gent/PS412, uses the MVB-derived exosomal pathway for efficient egress and spread in N2a cells.

**Fig 5:**
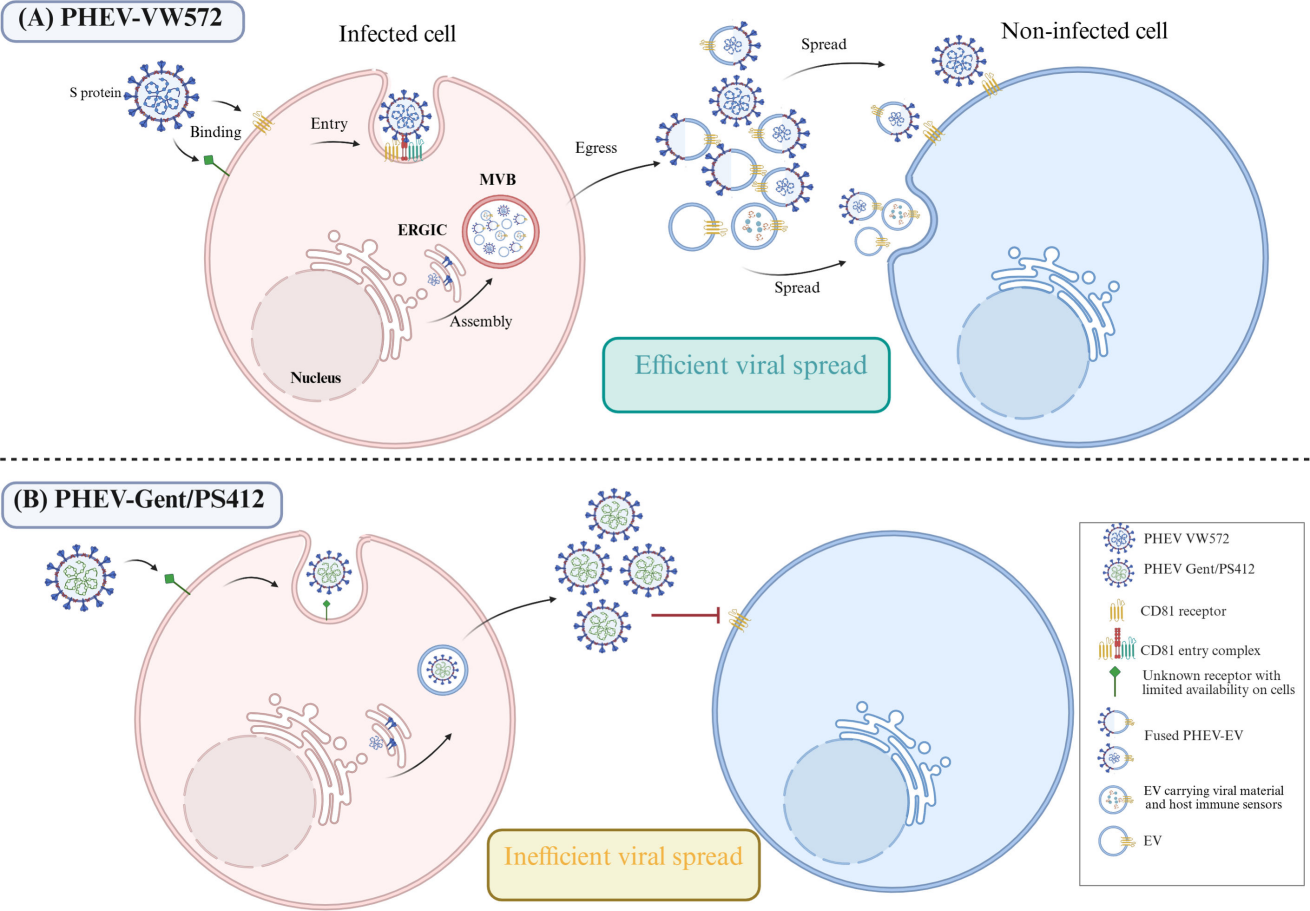
Hypothetical model of comparative PHEV life cycle into N2a cells between viral isolates. (**A**) The spike protein of PHEV-VW572 binds to the CD81 receptor on neuronal cells, and the interaction promotes receptor internalization by clathrin-mediated endocytosis. CD81 may work together with other tetraspanin(s) and protease(s) to form an entry complex and facilitate coronavirus entry, as seen for MERS-CoV. Alternatively, PHEV-VW572 binds to a receptor present on a restricted number of cells and enters by either fusion or endocytosis. PHEV-VW572 assembly occurs in the ERGIC, and virions hijack the MVB-derived exosomal pathway. MVBs contain mainly EVs and virus-fused EVs, as well as a few complete virions, which are simultaneously released by the infected cell after fusion of the MVB to the plasma membrane. Virions and virion-fused EVs can infect the next cell by interacting with the cellular CD81 receptor. In addition, EVs packaging viral material and proteins can be endocytosed by the uninfected cell, thus overall enhancing the infection. (**B**) PHEV-Gent/PS412 binds to a receptor present on a restricted number of cells and enters either by fusion or clathrin-mediated endocytosis. New progeny virions are assembled in the ERGIC and secreted from the infected cell by exocytosis. There is no hijack of the MVB-derived exosomal pathway and thus, only release of free virus particles to the extracellular environment. New virions do not bind to CD81 (only to the restricted receptor) and cannot further spread to neighboring cells.

### PHEV-VW572 uses exosomal marker tetraspanin CD81 as a receptor for entry into N2a cells

We previously showed that only 10% of neuronal cells were infected with PHEV at 24 hpi. Therefore, we hypothesized that a restriction at the binding and/or entry level(s) may occur in these cells. To test this, we characterized and compared the kinetics of PHEV binding to N2a cells between both isolates using Dio-labeled viral particles. As shown in Fig. 4A, a maximum of approximately 15%–20% of cells showed viral particles bound at their plasma membrane at 60 min post-binding, and this was true for both isolates. No significant difference in the number of bound virus particles (approx 2 particles/per cell) was observed between the two isolates at that time (Fig. 4B). Representative IF pictures of cells with bound PHEV particles are depicted in Fig. 4C. As viral particle aggregates were observed binding to cells for PHEV-VW572 by IF, we further characterized the entry mechanisms of PHEV by TEM. A picture of a PHEV virion is provided in Fig. 4D. As expected, the virion shows a spherical shape of approximately 81 nm in diameter with the presence of a helical nucleocapsid with a typical “crown-like” appearance. We found that the virus mainly enters cells via either engulfment via coated pits, indicative of clathrin-mediated endocytosis, or via direct fusion (Fig. 4E and F). While no major differences in entry mechanisms were observed between the two isolates by TEM, the presence of viral aggregates for VW572 may suggest a possible entry via multi-vesicular bodies. Overall, these findings indicate that PHEV binds to neuronal cells, independently of the viral isolate used.

Next, we determined if PHEV uses a specific receptor or set of receptors, present only on a restricted number of cells, for efficient entry, and if this strategy is shared by both isolates. The angiotensin-converting enzyme 2 (ACE2) has been identified as the main cellular receptor for SARS-CoV-2 (15). Still, N2a cells are known to express very low levels of ACE2, and transfection of these cells with an ACE2-GFP fusion plasmid is often required to study ACE2 cell localization and activity. In line with this, we did not observe a decrease in PHEV infection in cells treated with a function-blocking antibody against ACE2 compared to the untreated condition, independently of the isolate used (Fig. S6A, C, and D). As ß1 integrin has been proposed to serve as an alternative entry receptor for betacoronaviruses, we tested whether PHEV may similarly use it for entry into N2a cells (16). However, no significant decrease in PHEV infection was observed after blocking antibody treatment (Fig. S6B, E, and F). As PHEV-VW572 employs the MVB-exosomal pathway and releases fused PHEV-EVs into the extracellular environment, we tested whether it may use an exosomal receptor for entry into cells. Several tetraspanins, including CD81, CD63, and CD9, have been widely used as markers of exosomes for trafficking viral entry (17–19). Interestingly, CD81-enriched microdomains, along with CD9, are preferred entry sites for coronaviruses in neuronal cells (20, 21). Pretreatment of cells with anti-CD81 blocking antibody significantly inhibited PHEV infection of neuronal cells at 24 hpi, though only for the VW572 isolate (Fig. 4G). Comparative IF pictures of PHEV infection between both isolates and upon CD81 antibody treatment are provided in Fig. 4H and I. Finally, we tested whether small interfering RNA (siRNA) targeting CD81 could efficiently reduce PHEV infection in neuronal cells. At 24 h post-transfection with siRNA sequences, including CD81-targeting and non-targeting control siRNA, cells were inoculated with PHEV for 24 h, and viral protein expression was assessed by IF staining. We demonstrated a significant decrease in PHEV infection in CD81 siRNA-treated cells compared to non-treated ones (Fig. S7). Overall, these findings confirmed that PHEV-VW572 entry into neuronal cells is mediated by the CD81 receptor.

## DISCUSSION

Neuroinfectious diseases represent a major threat to public health worldwide. Respiratory viruses, such as betacoronaviruses, have been shown to be associated with a broad range of acute, as well as chronic and long-term neurological manifestations (22–25). To date, the pathophysiological mechanisms associated with these viral infections remain poorly understood. Using PHEV, a surrogate model to study betacoronaviruses, we investigated and compared key steps in the replication cycle of three distinct viral isolates in mouse neuronal cells.

First, we compared the replication kinetics of these isolates and demonstrated that viral infection is restricted at early time points in neuronal cells compared to control porcine kidney cells (RPD). No significant increase in the percentage of infected cells was observed for both PHEV-Gent/PS412 and -Labadie isolates throughout the course of infection. In contrast, PHEV-VW572 seemed to overcome the early restriction as demonstrated by an increased percentage of infected cells at 48 hpi. These results suggest that PHEV-VW572 replicates more efficiently in neuronal cells than PHEV-Gent/PS412 and -Labadie isolates. These results also correlate with the clinical outcome of the infection in pigs. The Belgian PHEV-VW572 was isolated from a pig with vomiting and wasting disease. The presence of infectious PHEV antigen was confirmed in the trigeminal and vagal ganglia following oronasal inoculation in pigs (8). In contrast, PHEV-Gent/PS412 was isolated from a pig that showed respiratory but no neurological symptoms. This may explain why this isolate does not replicate efficiently in neurons. For the PHEV-Labadie isolate, limited information was provided about its isolation from a French farm. Therefore, we performed a complete genomic characterization of this isolate, and a phylogenetic tree was constructed based on the whole-genome sequences of PHEV-VW572, -Gent/PS412, and -Labadie (Fig. S8). The phylogenetic analysis shows that there is a closer relationship between PHEV-Gent/PS-412/2020 and VW572, while the PHEV Labadie is more divergent from those two. The closest relatives for Gent/PS-412/2020 are LJ/2021 and VW572. There is more genetic divergence between isolate Labadie and PHEV-Gent/PS-412/2020, as illustrated by the branch lengths in the tree. This is in contrast to our data showing similar replication kinetics between PHEV Labadie and Gent/PS412 in N2a cells. It is possible that PHEV-Labadie restricts viral expression and replication in neurons as a strategy for viral persistence, a common feature shared among other coronaviruses (26–28). A comparative *in vivo* neuropathogenesis study of these isolates in pigs is needed to further investigate this process.

Second, we demonstrated that PHEV-VW572 infection resulted in a high intracellular virus titer alongside an extracellular virus titer, which did not increase. We initially proposed that new progeny virions accumulated inside the cells upon infection as part of an immune-evasive strategy for efficient viral spread. However, we found that neutralizing antibodies abrogated PHEV infection in N2a cells, suggesting that the virus spreads extracellularly rather than cell-to-cell. These results are in striking contrast with previous literature showing that SARS-CoV mediates neuronal cell-to-cell spread to enhance its neuroinvasiveness (11). In recent years, exosomes have been reported to play an important role in facilitating betacoronavirus transmission and modulating host immune responses. Exosomes are small extracellular vesicles (EVs) that are released into the extracellular space when the plasma membrane fuses with an MVB formed via endocytosis. These vesicles are classified based on their size, which can range from 30 to 100 nm (exosomes) to 100–1,000 nm (microvesicles) (29). Upon treatment with an inhibitor of exosome biogenesis (GW4869), we demonstrated that PHEV-VW572, but not Gent/PS412 isolate, uses the MVB-derived exosomal pathway for viral egress in neuronal cells. These results were further confirmed by TEM, where the presence of PHEV-VW572 virions was seen within intracellular vesicles. Nevertheless, no clear signs of exocytosis or release of virions within EVs were seen at the cell surface. In contrast, we observed the presence of fused PHEV-EV structures close to the plasma membrane. These results are partially in agreement with the study from Li et al., which demonstrated the presence of PHEV-modified exosomes in supernatants of PHEV-infected cells by EM (30). The authors concluded that PHEV mainly uses exosomes for cell communication, specifically for mediating the transfer of immunostimulatory cargo to uninfected neuroimmune cells. Still, this study only included one PHEV strain (PHEV-CC14), and the authors failed to generalize their conclusions to other PHEV strains or isolates. In contrast, we demonstrated that PHEV assembly and egress are isolate-dependent, and PHEV-VW572 uses the MVB-derived exosomal pathway as a strategy to promote efficient infection and overcome the early restriction in N2a cells. Notably, the inhibitor GW4869 resulted in a significant but partial reduction (approx 50%) in PHEV infection, therefore suggesting the involvement of alternative viral dissemination mechanisms independent of the GW4869-sensitive pathway.

Third, we showed that PHEV binds to approximately 15%–20% of N2a cells, independently of the PHEV isolate used. These results suggest that the restricted PHEV infection observed at the early stage of infection (24 hpi) may occur at a binding level. Betacoronavirus entry is initiated by the binding of viral spike (S) protein to a cellular receptor, followed by proteolytic cleavage of S protein and release of the fusion peptide, allowing for host-cell entry (31). As the use of specific cell receptors is often responsible for viral tropism, we screened potential receptor candidates for PHEV entry into cells by performing function-blocking antibody experiments. Blocking ACE2 and ß1 integrin receptors had no effect on viral infection, suggesting that these two receptors are not involved in PHEV entry into N2a cells. For ACE2, this is not surprising as very low levels of mRNA and protein expression have been detected in these cells (32, 33). In contrast, a study from Lv et al. (34) showed that ß1 integrin mediates PHEV entry into cells. It is important to note that the authors used a different viral isolate than those examined in our current study. This underlines again the isolate-specific differences in PHEV pathogenesis. Interestingly, we demonstrated that PHEV-VW572, but not PHEV-Gent/PS412 isolate, uses the tetraspanin CD81 receptor for entry into N2a cells. Tetraspanins play a crucial role in this process by acting as scaffolding proteins that facilitate coronavirus infections by forming specialized microdomains on cell membranes. For instance, it was shown that MERS-CoV enters cells using an entry complex that includes a receptor, a protease, and CD9 tetraspanin (18). The inhibition of PHEV-VW572 infection following neutralizing antibody or siRNA treatment suggests that CD81 is the main receptor for the virus in neuronal cells. The exploitation of tetraspanin-enriched microdomains for efficient cell entry has also been observed for other viruses, such as HIV-1 (35). Additionally, the enrichment of CD81 at the cell membrane and its colocalization with structural proteins in neuronal cells may explain why it serves as a preferred entry site for PHEV (36). The fact that the CD81 receptor is not involved in the entry of PHEV-Gent/PS412 isolate further confirms that isolate-specific differences exist in cellular receptor use. Strikingly, CD81 is the most highly enriched protein in EVs, even though it is primarily localized inside the plasma membrane (37). In accordance with the literature, we therefore hypothesize that CD81 may also be expressed on fused PHEV-EVs and facilitate exosomal fusion with the target cell, thus enhancing the overall infection (38). This mechanism may explain why PHEV-VW572 can overcome the early block of infection and efficiently spread in N2a cells over time, while PHEV-Gent/PS412 is not able to do so. A hypothetical model of PHEV entry and spread into N2a cells is provided in Fig. 5. Finally, we showed that both isolates could enter N2a cells either by direct fusion at the plasma membrane or by clathrin-mediated endocytosis, as shown by TEM. These results are consistent with previously reported mechanisms for PHEV (39). Still, it is important to mention that accurate identification of coronavirus particles by TEM remains challenging due to morphological similarities with subcellular structures. Clathrin-coated vesicles have a size similar to coronavirus, but lack nucleocapsid cross-sections and occur freely in the cytoplasm rather than within vacuoles (40, 41). These diagnostic pitfalls highlight the necessity of stringent morphological criteria to differentiate coronaviruses, such as PHEV, from cellular mimics in TEM studies. In conclusion, this study reveals new mechanisms by which PHEV efficiently infects and spreads within neuronal cells. It may also contribute to furthering our understanding of betacoronavirus neuropathogenesis in order to develop new treatments against neurological manifestations. 

## MATERIALS AND METHODS

### Cells

Porcine kidney cells (Rein de Porc, or RPD) were maintained in RPMI glutamax medium with 10% fetal calf serum, 1% streptomycin, and 0.5% gentamicin, and incubated at 37°C with 5% CO_2_. Murine neuroblastoma (N2a) cells (ATCC CCL-131) were cultured in Dulbecco’s modified Eagle medium (DMEM) with supplements and incubated at 37°C with 5% CO_2_.

### Virus

PHEV-VW572 (accession no. DQ011855) (stock titer of 10^6,8^ TCID_50_/mL) was isolated in Belgium in 1972 from the tonsils of two diseased pigs suffering from vomiting and wasting disease (42). PHEV-Labadie (accession no. PV820711.1) (stock titer of 10^6.3^ TCID_50_/mL) was isolated by veterinarian JL Labadie in the 1970s from a diseased pig in a farm in France. PHEV-Gent/PS412 (accession no. PV820712) (stock titer of 10^6,96^ TCID_50_/mL) was isolated from a pig with only respiratory symptoms in Belgium in 2020 and sequenced by Pathosense (Ghent University).

### Viral inoculation

Cells were (mock) inoculated with either PHEV-VW572, Gent/PS412, or PHEV-Labadie isolates at an MOI of 1 (or 5 when indicated) and incubated for 1 h at 37°C with 5% CO_2_. After washing with warm DMEM (N2a), 1 mL of culture medium was added, and the cells were further incubated at 37°C with 5% CO_2_. Cell viability was checked by propidium iodide (10 µg/mL, Hello Bio) and trypan blue (0.4%, ThermoFisher) staining. Cell viability was >90%. At corresponding time points, the cell supernatant and lysate were collected and stored at −80°C for viral titration. Cells were fixed with 4% PFA for 10 min, washed with phosphate-buffered saline (PBS), and stored in PBS at 4°C for immunofluorescence (IF) staining.

### Virus titration

To quantify the PHEV infectious virus, both intracellular and extracellular virus titers were determined. The supernatant and cell lysate were collected at different hpi. Viral titration was performed on RPD cells, which are known to be fully susceptible to PHEV infection. The virus titer was calculated as 50% tissue culture effective dose (TCID_50_) according to the Reed and Muench formula (43).

### Genomic RNA quantification

Total intracellular and extracellular genomic RNA was extracted using the RNeasy Mini Kit (QIAGEN) and the IndiSpin Pathogen Kit (Indical Bioscience), respectively. PHEV N gene RNA was quantified by reverse transcription quantitative PCR using the Takyon One-Step Kit Converter with previously described PHEV N gene-specific primers (7). A standard curve was generated using serial dilutions of a purified 334 bp amplicon of the PHEV-N gene, and viral RNA copy numbers were calculated and expressed as copies/μL. Amplification was performed with Takyon Low Rox SYBR MasterMix dTTP Blue (Eurogentec) on a QuantStudio 3 Real-Time PCR System (Thermo Fisher Scientific).

### Antibodies

The following primary antibodies were used at optimized concentrations: swine polyclonal anti-PHEV biotinylated (1/20, produced in-house), swine anti-PHEV serum (0.86 and 2.15 mg/mL, produced in-house), polyclonal IgG CD81 (Novus Biologicals, NBP2-20564), monoclonal IgG1 anti-integrin ß1 antibody (Abcam, ab24693), and ACE2 antibody polyclonal IgG (Bio-Techne, AF933). The following secondary antibodies were used: streptavidin-FITC (1/200, ThermoFisher Scientific), streptavidin-TR (1/200, ThermoFisher Scientific), goat anti-rabbit AF594 (1/200, ThermoFisher Scientific), and goat anti-rabbit AF488 (1/200, ThermoFisher Scientific).

### IF staining and microscopy

Fixed cell coverslips were washed with PBS and permeabilized using 0.1% Triton X-100 for 2 min, followed by two additional washes with PBS. For staining with PHEV biotinylated antibody, an extra step was performed in which cell coverslips were pre-incubated with an avidin/biotin solution (ThermoFisher Scientific) for 15 min at 37°C to reduce non-specific binding due to endogenous biotin. Cell coverslips were incubated for 1 h at 37°C with appropriate primary antibodies diluted in 10% negative goat serum and PBS. Appropriate isotype-matched controls were included. Coverslips were washed three times with PBS and incubated for 50 min at 37°C in the dark with appropriate secondary antibodies diluted in PBS only. The nuclei were counterstained with Hoechst 33342 (10 μg/mL, ThermoFisher Scientific) for 10 min at 37°C. Coverslips were rinsed three times with PBS and once with ultra-pure (UP) water. Coverslips were mounted with glycerol-DABCO (ACROS Organics, USA). Fluorescent and confocal microscopy were carried out using Leica Microsystems DMRBE and a confocal laser scanning microscope from ZEISS (ZEISS LSM 900, Germany), respectively.

### Transmission electron microscopy

Cells were gradually fixed *in situ* by adding 2.5% glutaraldehyde in 0.05 M sodium cacodylate buffer (EMS #11654) to the culture medium at a 1:1 vol ratio, incubated at 37°C for 5 min. The final 1:0 vol ratio was incubated at 37°C for 20 min, followed by 40 min at room temperature (RT). After fixation, cells were washed four times with 0.1 M sodium cacodylate buffer. Cells were post-fixed using 1% osmium tetroxide (EMS #19150) for 1 h at RT. Cells were subsequently washed four times with UP water, and an en bloc staining step was performed using 1% uranyl acetate (EMS #22400-1) in UP water at 4°C for 1 h in the dark, followed by four washes. Cells were slowly dehydrated in a graded ethanol series for 15 min each. The dehydrated samples were then infiltrated in a 2:1 vol ratio mixture of 100% ethanol and Spurr resin (EMS #14300) for 2 h at RT, followed by a 1:2 vol ratio mixture and fully infiltrated with 100% Spurr resin overnight at 4°C. The next day, two additional changes of Spurr resin were made, ending with a final 100% infiltration overnight at 4°C. The coverslip samples were embedded in fresh Spurr resin and polymerized at 60°C for a minimum of 8 h.

Ultrathin sections (~70 nm) were cut using a Leica EM UC6 ultramicrotome equipped with a diamond knife (Ultra 45°, 2.5 mm DiATOME #DU4525). Sections were collected on copper formvar support single slot grids (EMS FF2010-Cu-50) and stained with 1% uranyl acetate at 37°C for 30 min in the dark, followed by a rinse, and further stained once dried with lead citrate for 5 min at RT, followed by a final rinse. Once dried, the sections were examined using a transmission electron microscope (JEOL JEM-1400 plus BF-TEM). Digital images were captured with a Quemesa device camera (Olympus Soft Imaging Solutions, Germany) at various magnifications to visualize subcellular structures and any virus-like particles or organelles of interest.

### Antibody neutralizing assay

To determine whether PHEV can spread cell-to-cell, PHEV-inoculated cells were incubated with swine anti-PHEV neutralizing antibody serum for 48 h at 37°C with 5% CO_2_. Cell coverslips were fixed with 4% PFA for 10 min, and IF staining was performed, as previously described.

### Neutral sphingomyelinase inhibitor GW4869

Cells were pretreated with GW4869 (10 µM) for 24 h, followed by inoculation with PHEV at an MOI of 1 for 1 h. When indicated, cells were first inoculated with PHEV at an MOI of 5, the inoculum was removed, and cells were washed before adding the inhibitor. After 48 h of incubation, cells were fixed and stained for PHEV S protein, as described above.

### PHEV purification and Dio-labeling

Viral purification was performed as previously described (44). Purified PHEV-VW572 and -Gent/PS412 isolates were labeled with Dio (1:100, Vybrant DiO Cell-Labeling, Solution Thermo Fisher) dissolved in DMSO (Molecular Probes), by vigorous vortexing followed by a 1 h incubation at RT. After filtration using a Sephadex G-50 column, the infectivity of Dio-labeled virus remained significantly unchanged. The purity of the PHEV suspensions was assessed using lipophilic labeling and IF staining. Confocal microscopy was used to analyze the staining by randomly selecting five regions.

### PHEV binding assay

Cells were cooled down on ice for 5 min and inoculated with 250 µL of Dio-labeled VW572 and Gent/PS412 virus at an MOI of 1 for 1 h on ice. Afterward, cells were washed twice with cold DMEM to remove unbound particles and were further incubated for 5, 10, 15, 30, and 60 min. Cell coverslips were fixed with 1% PFA for 10 min at 37°C and counterstained with Hoechst. The percentage of PHEV-positive cells was determined by counting the number of cells with viral particles bound on the plasma membrane of 15 randomly selected cells for each isolate. The mean number of virus particles bound per cell was calculated based on the number of virus particles attached to the plasma membrane of five randomly selected PHEV-positive cells using z-stack imaging.

### Antibody blocking assay

Cells were pre-incubated for 2 h at 37°C with the following function-blocking antibodies: polyclonal IgG anti-CD81 (5 µg/mL, Novus Biologicals, NBP2-20564), monoclonal IgG1 anti-integrin ß1 (5 µg/mL, Abcam, ab24693), polyclonal IgG anti-ACE2 (5 µg/mL, Bio-Techne, AF933), or appropriate isotype controls. After washing, cells were inoculated with PHEV for 1 h, as described above. Function-blocking antibodies were further kept in the medium for 48 h at 37°C. IF staining was performed as previously described.

### siRNA transfection

N2a cells were seeded into a 24-well plate one day before transfection. Cells were transfected with CD81-targeting siRNA (Ambion) at a final concentration of 100 nM using Lipofectamine RNAiMAX (Invitrogen), following the manufacturer’s instructions. The siRNA sequences used were sense: 5′-GUACCUCAUUGGAAUUGCAtt-3′, antisense: 5′-UGCAAUUCCAAUGAGGUACag-3′. The 5′-AATCGGGCAGTTGTTTGAGAT-3′ (siCRK) sequence, corresponding to positions 1,023 to 1,043 of Leishmania CRK1, a protein kinase, was chosen as a negative control. siRNA and lipid-based transfection reagent were diluted in Opti-MEM, incubated to allow complex formation, and then added to the cells in complete growth medium. After 24 h of transfection, cells were inoculated with PHEV-VW572 at an MOI of 1. Cells were fixed at 24 hpi, and IF staining was performed, as previously described.

### Statistical analysis

Data were analyzed with GraphPad Prism 9.3.0 software (GraphPad Software Inc.). For statistical significance, data were subjected to a one-way or two-way analysis of variance (ANOVA) followed by a *t*-test and Tukey’s multiple comparisons, respectively. All presented results represent the means and standard deviation (SD) of three independent experiments. *, *P* value < 0.05; **, *P* value < 0.01; ***, *P* value < 0.001; ****, *P* value < 0.0001; ns, not significant.

## Data Availability

All data supporting the findings of this study are available within the article and its supplemental material. Additional data are available from the corresponding author upon reasonable request. The genome sequences generated in this study have been deposited in the GenBank database under accession numbers DQ011855, PV820711.1, and PV820712.

## SUPPLEMENTAL MATERIAL

Supplemental figures jvi.01171-25-s0001.docx Figures S1 to S8.

ASM does not own the copyrights to Supplemental Material that may be linked to, or accessed through, an article. The authors have granted ASM a non-exclusive, world-wide license to publish the Supplemental Material files. Please contact the corresponding author directly for reuse.
