# Supplementary material for: Porcine hemagglutinating encephalomyelitis virus VW572 (not Gent/PS412 and Labadie) uses the CD81 receptor and MVB-derived exosomal pathway for efficient entry and spread in neuronal cells
Source: J Virol. 2025 Oct 8;99(11):e01171-25. doi: 10.1128/jvi.01171-25 (PMC12645993; doi:10.1128/jvi.01171-25)
Supplement: Supplemental figures — Figures S1 to S8. [file jvi.01171-25-s0001.docx]

**Supplementary Figures**


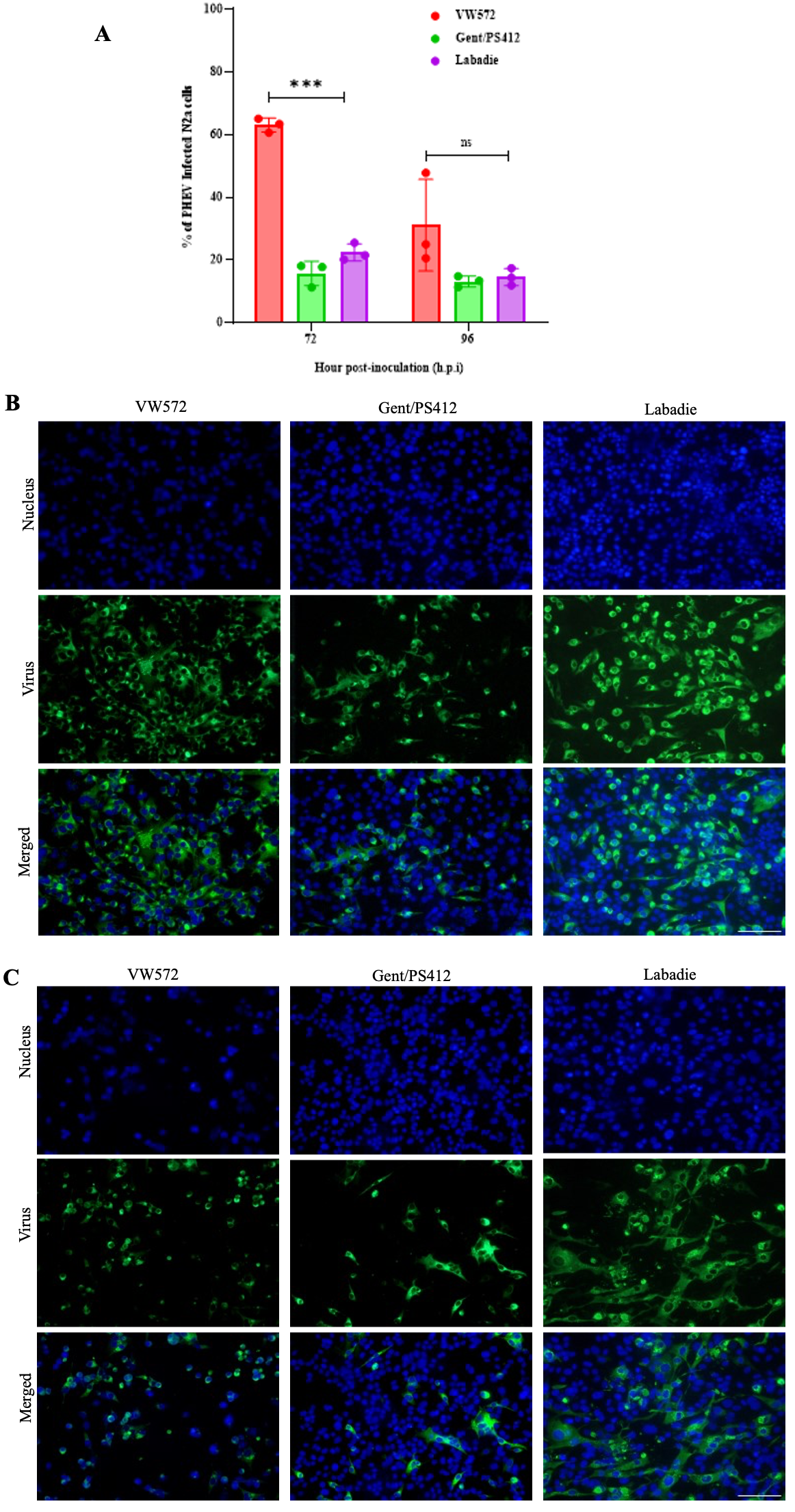


**Figure S1. Comparative replication kinetics of PHEV isolates in N2a cells at 72 and 96hpi.**

(A) Kinetics of PHEV protein expression in N2a cells. Cells were (mock) inoculated at a MOI of 1 with either PHEV VW572, Gent/PS412 or Labadie. The percentage of PHEV-infected cells was quantified at 72 and 96hpi by IF staining. Error bars indicate standard deviation (SD) and ns, not significant; ***, p < 0.001. (B) and (C) Representative IF images of PHEV infected cells at corresponding time points. Fixed cells were stained for PHEV S protein (green) and nuclei were counterstained with Hoechst 33342 (blue). Scale bar represents 100µm.


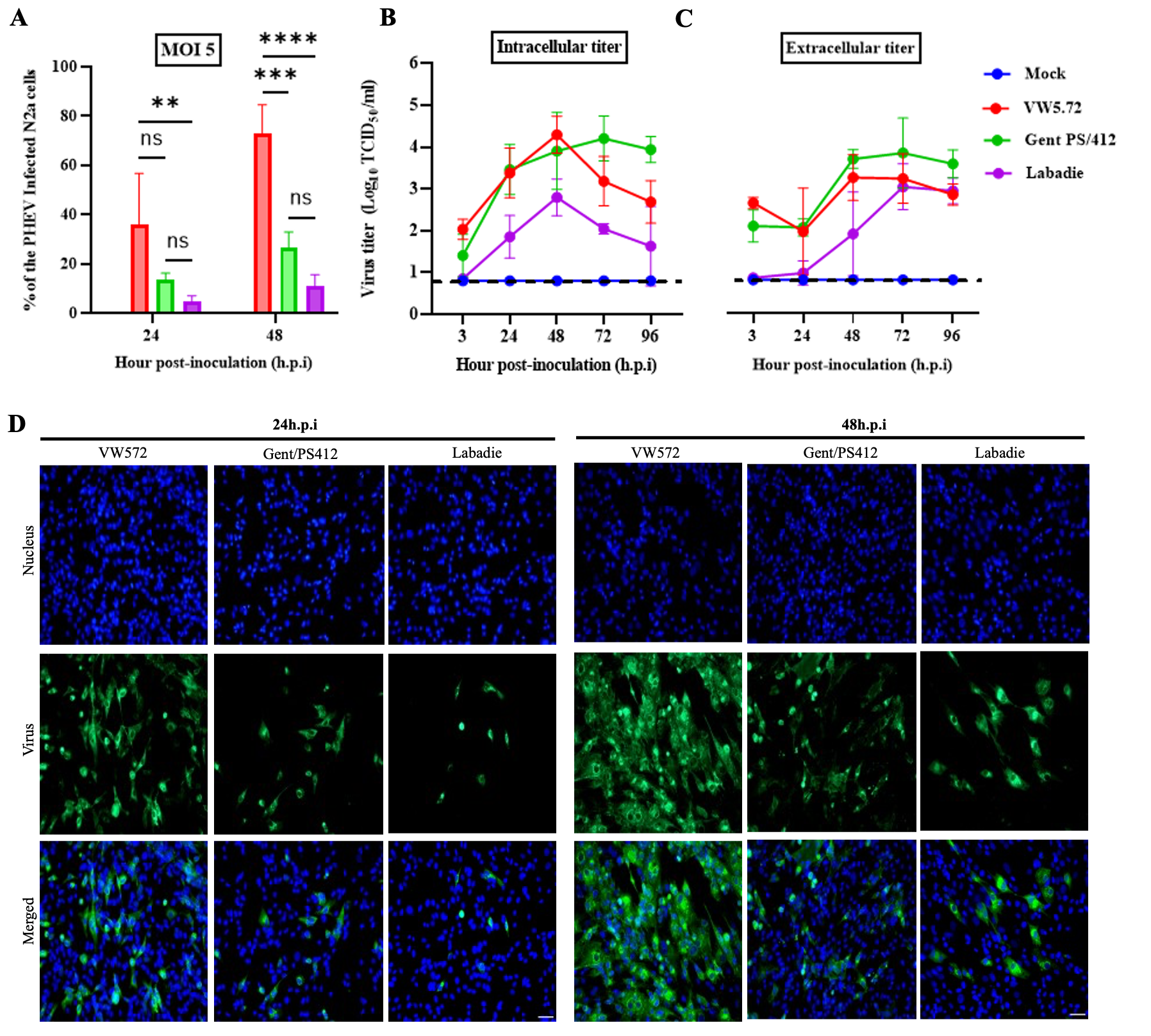
**Figure S2. Comparative replication kinetics of PHEV isolates in N2a cells at a MOI of 5.**

(A) Kinetics of PHEV protein expression in N2a cells. Cells were (mock) inoculated at an MOI of 5 with either PHEV-VW572, -Gent/PS412, or -Labadie. The percentage of PHEV-infected cells was quantified at 24 and 48hpi by IF staining. Error bars indicate standard deviation (SD) and ns, not significant; **, p< 0.01; ***, p < 0.001; **** p< 0.0001. (B) and (C) Kinetics of intracellular and extracellular virus titers in N2a cells. Virus titers are expressed as log_10_ TCID_50_/ml. (D) Representative IF images of PHEV-infected cells at 24 and 48hpi. Fixed cells were stained for PHEV S protein (green), and nuclei were counterstained with Hoechst 33342 (blue). Scale bar represents 50µm.


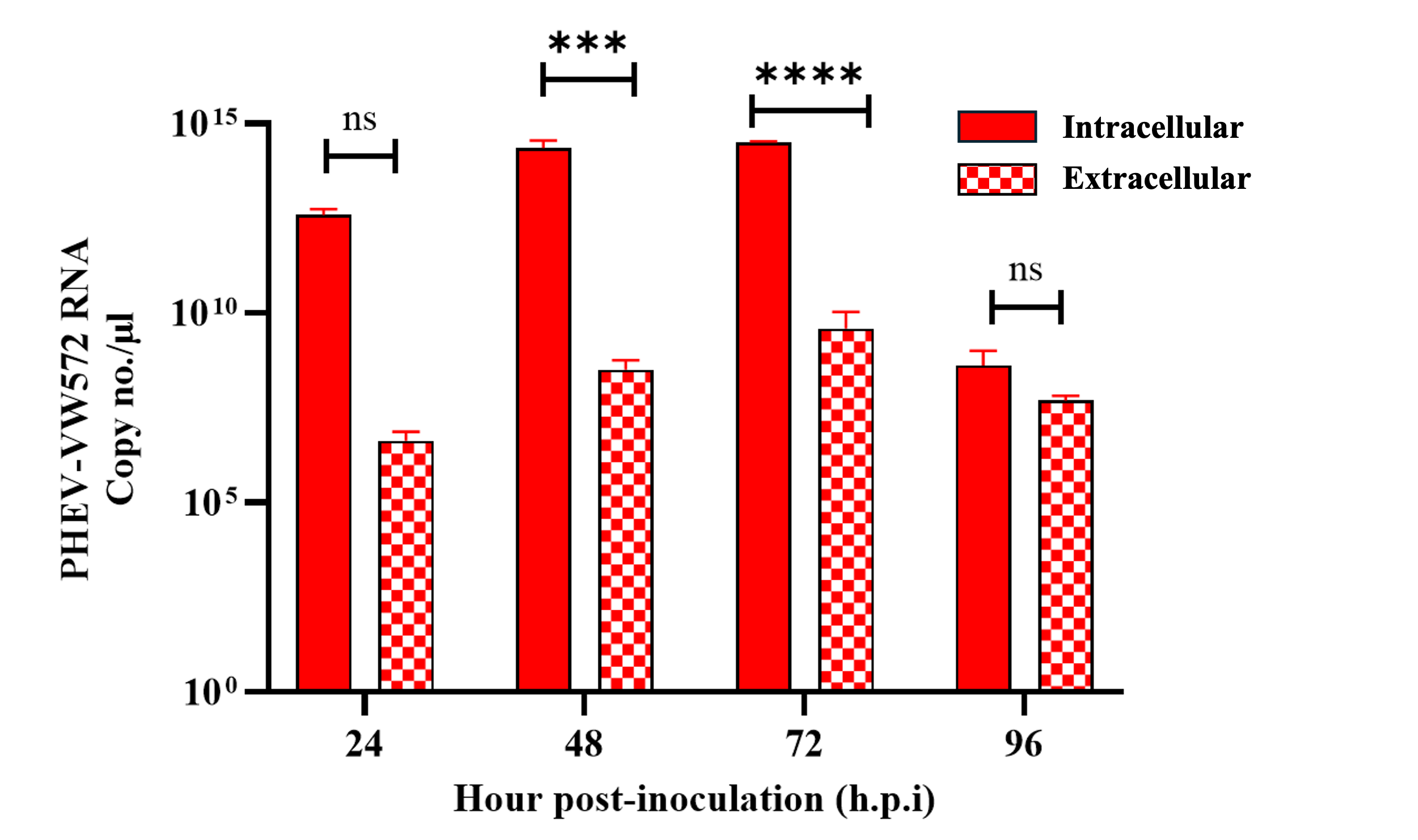


**Figure S3.** **Quantification of PHEV genomic RNA in extracellular and intracellular compartments.**

The levels of PHEV-VW572 genomic RNA were determined by quantitative real-time PCR (qPCR) in extracellular (culture supernatant) and intracellular (cell-lysate) fractions of PHEV-VW572-infected N2a cells at 24, 48, 72, and 96 hours post-inoculation (h.p.i). Data were normalized to the RNA copy number by constructing a standard curve. The RNA copy number (copies/μl) is plotted on a logarithmic scale (base 10). Data are presented as mean ± standard deviation (SD) from three independent experiments.


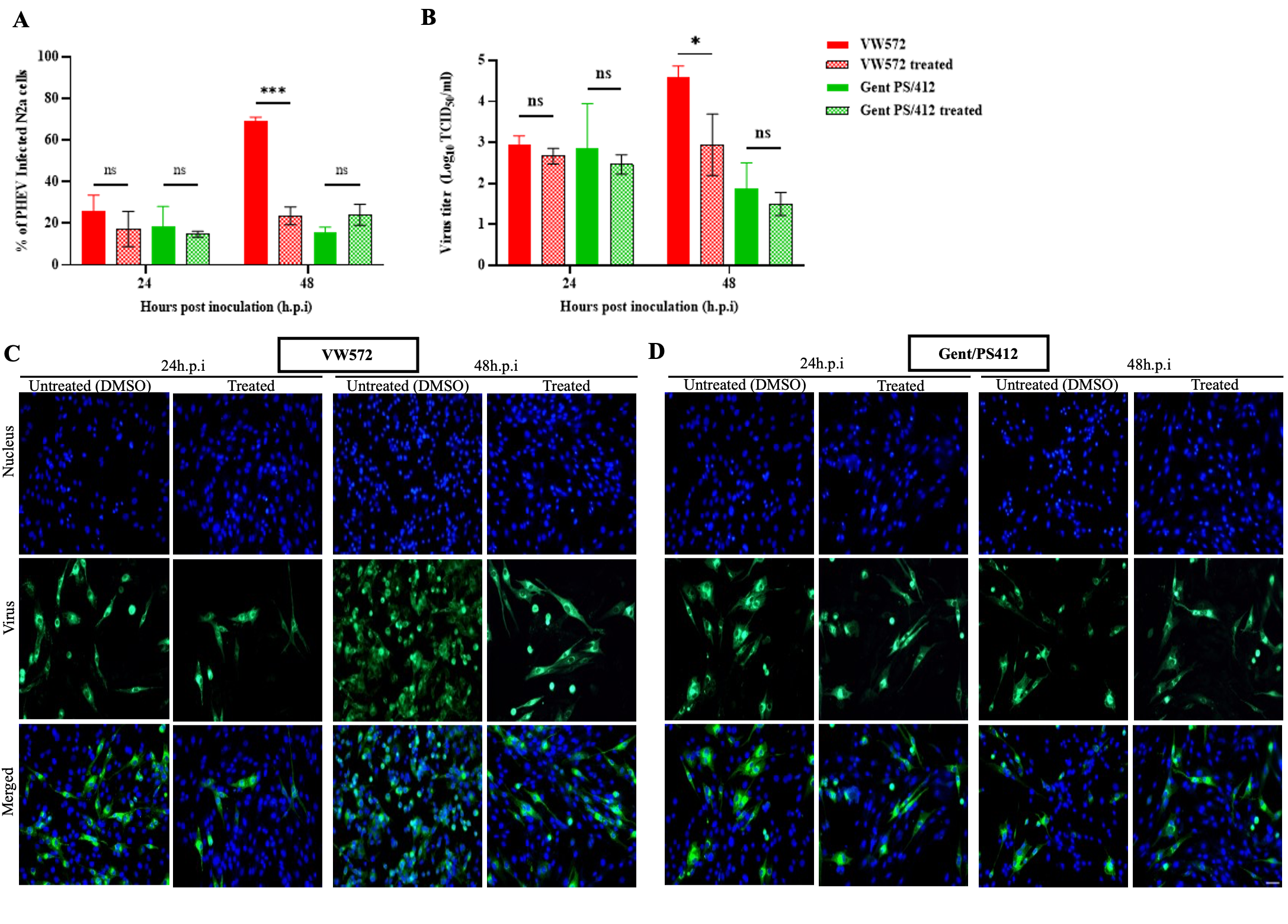


**Figure S4.** **GW4869 inhibitor does not affect the initial entry of the virus particles.**

(A) Percentage of PHEV-infected N2a cells at MOI 5 and (B) Quantification of PHEV extracellular titers (log_10_ TCID_50_/ml) following treatment with GW4869 (10 μM) or control (DMSO) after the 1h viral incubation period. Error bars indicate standard deviation (SD) and ns, not significant; *, p < 0.05; ***, p < 0.001. (C) and (D) Representative IF images of PHEV-VW5.72 and -Gent/PS412-infected N2a cells upon GW4869 treatment at 24 and 48 hpi. Cells were stained for PHEV S protein (green) and nuclei were counterstained with Hoechst 33342 (blue). Scale bars represent 50µm.


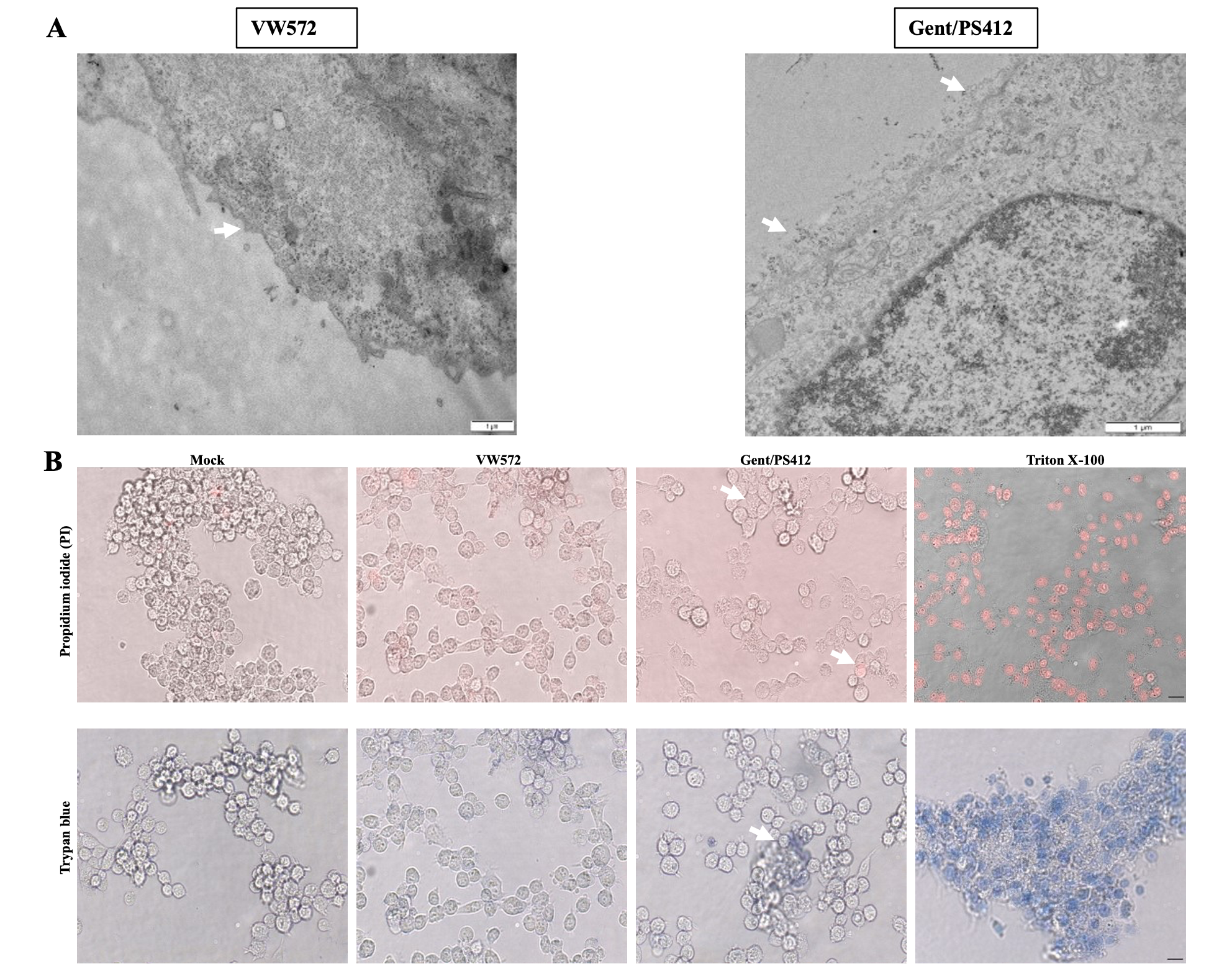


**Figure S5. Assessment of ultrastructural membrane changes and membrane permeability in PHEV-infected N2a cells.**

(A) Transmission electron microscopy (TEM) analysis of PHEV-infected N2a cells at 24hpi revealed distinct differences in membrane integrity between the two viral isolates. Cells infected with the VW572 isolate (left) exhibited intact plasma membranes. In contrast, Gent/PS412-infected cells (right) displayed notable disruptions and structural damage to the membrane, as indicated by white arrows. Scale bar represents 1µm. (B) Assessment of cell membrane permeability and viability in PHEV-infected N2a cells using propidium iodide (PI, top panels) and trypan blue exclusion (bottom panels). Cells treated with Triton X-100 were used as a positive control. PI-positive (red-stained) nuclei and trypan blue uptake indicate more compromised membrane integrity in Gent/PS412-infected cells exhibit greater membrane permeability (white arrows), relative to VW5.72. Scale bar represents 20µm.

**
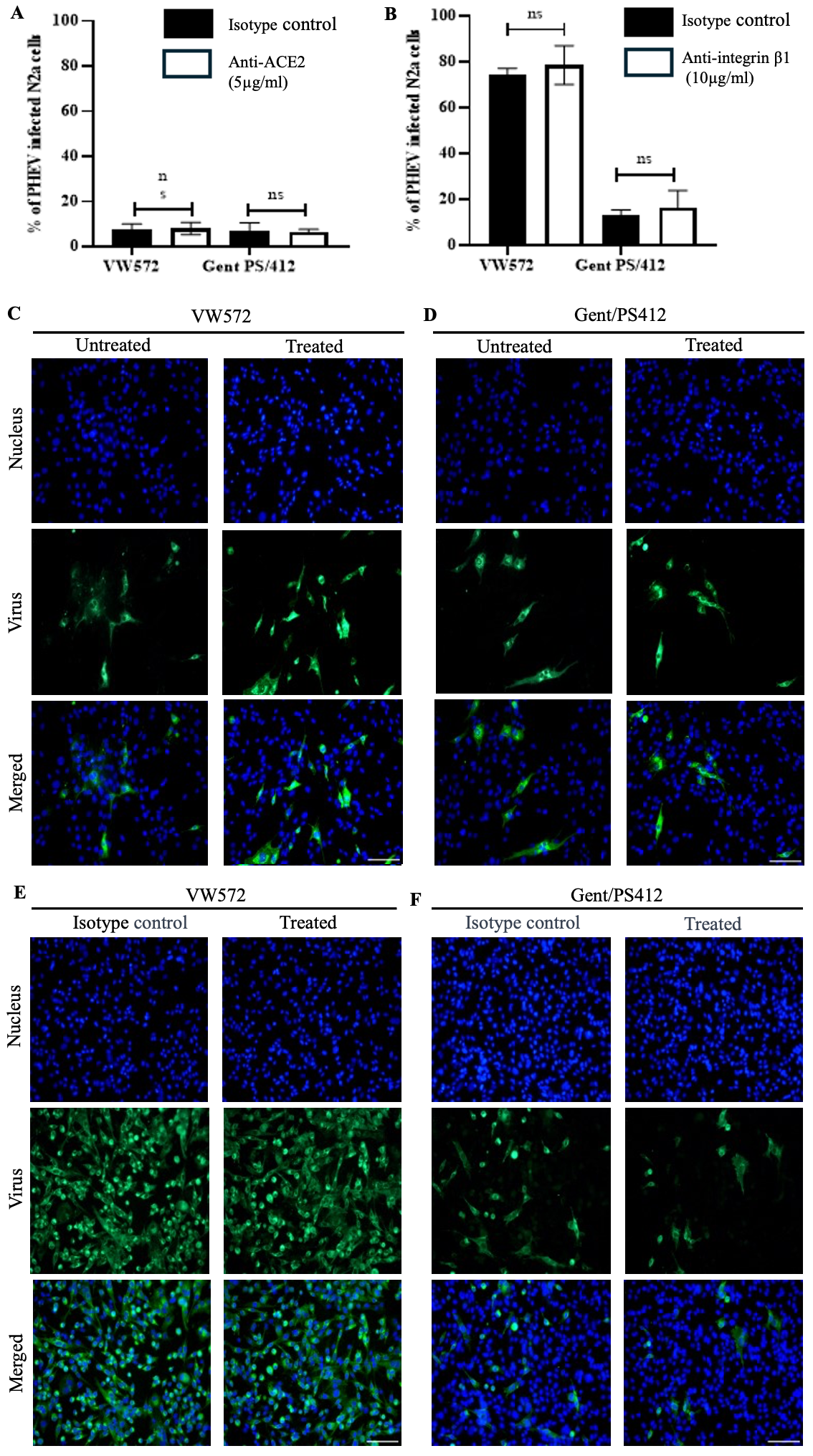
**

**Figure S6. Quantification of PHEV infection in N2a cells following pretreatment with blocking antibodies against ACE2 and integrin β1.**

(A) and (B) Percentage of PHEV-infected N2a cells following pretreatment with anti-ACE2 and anti-integrin β1 blocking antibodies, respectively. Cells were pretreated with blocking antibodies or appropriate isotype control for 2h at 37°C prior to inoculation with PHEV-VW572 or Gent/PS412 isolates at a MOI of 1. Antibodies were kept in the medium until cell fixation at 24 or 48hpi. Error bars indicate standard deviation (SD) and ns= not significant. (C) and (D) Representative IF pictures showing PHEV infection in anti-ACE2-treated versus control conditions at 24hpi. (E) and (F) Representative IF pictures showing PHEV infection in anti-integrin β1-treated versus control conditions at 48hpi. Cells were stained for PHEV S protein in green and nuclei were counterstained in blue. Scale bars represent 100µm. ns= not significant.


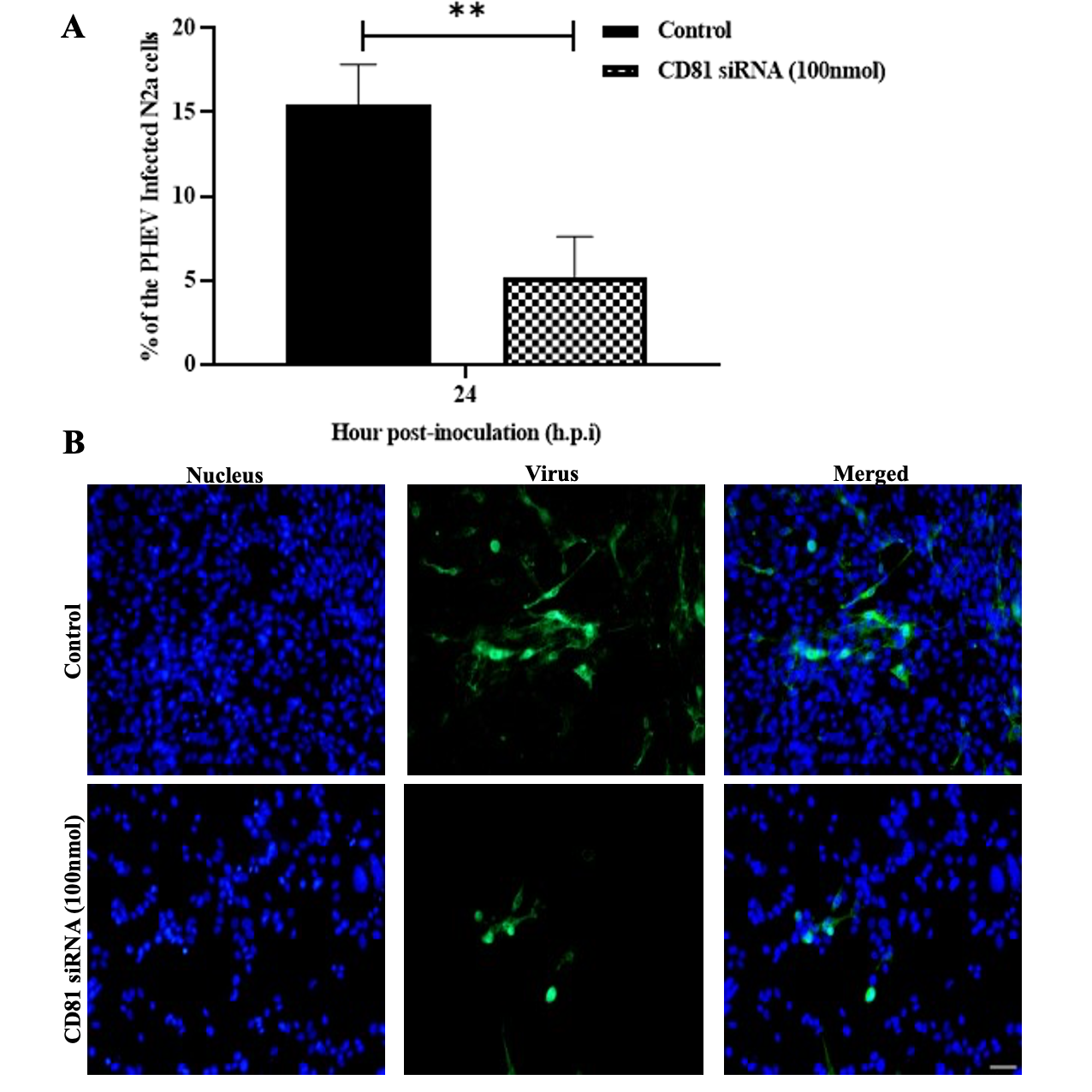


**Figure S7. CD81 siRNA knockdown reduces PHEV infection in N2a cells.**

(A) Percentage of PHEV-infected N2a cells following siRNA treatment. Cells were treated with 100nmol of siRNAs for 24h and then inoculated with PHEV VW572 at a MOI of 1 for 24h. Error bars indicate standard deviation (SD) and **, p< 0.01. (B) Representative IF pictures showing PHEV infection in CD81 knockdown versus non-targeting control conditions at 24hpi. Scale bar represents 25µm.


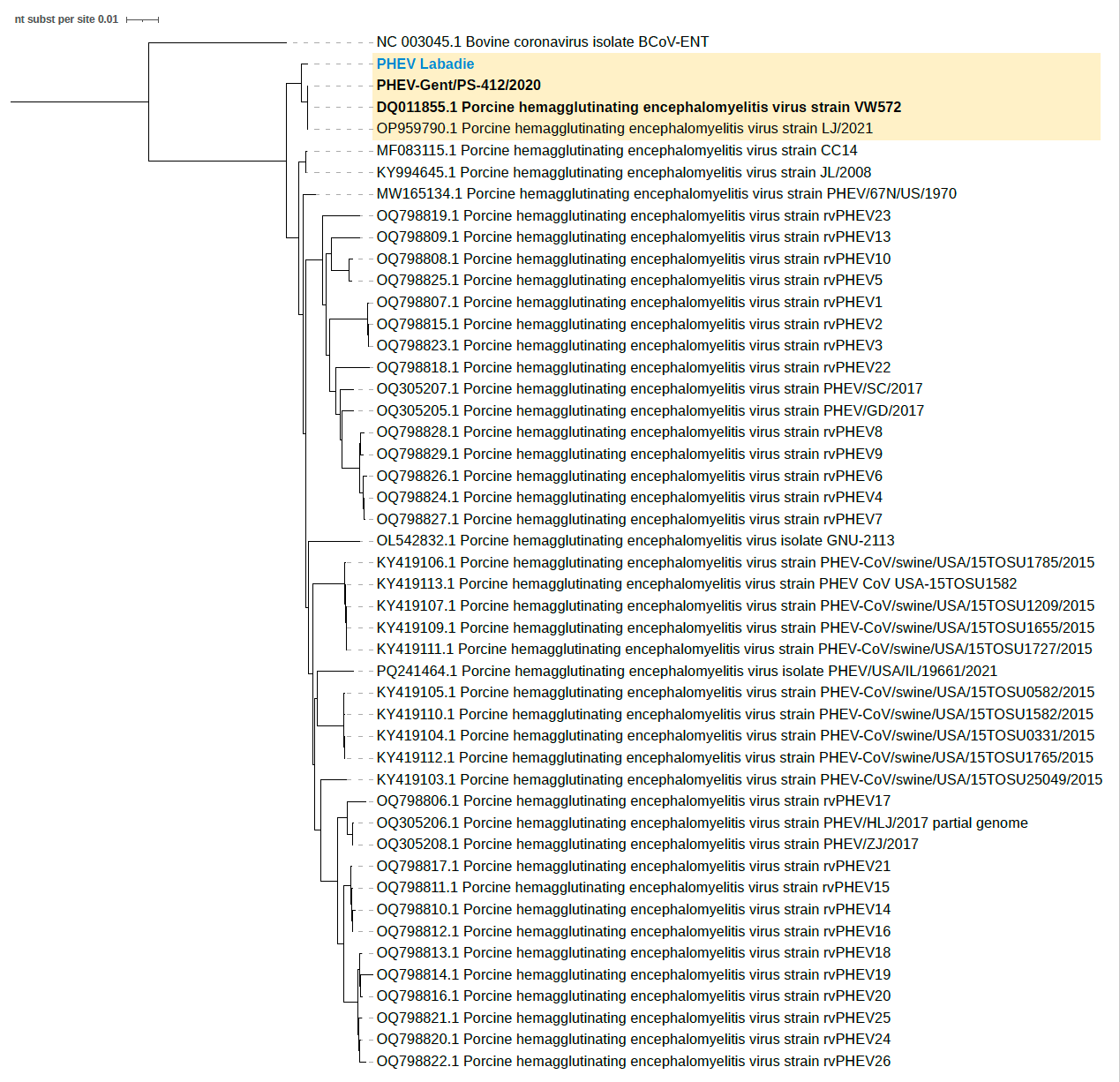
**Figure S8. Phylogenetic positioning of PHEV Labadie compared to known PHEV isolates.**

A phylogenetic analysis was conducted for PHEV Labadie isolate using the available porcine hemagglutinating encephalomyelitis virus whole genome sequences from NCBI (n=45), supplemented with the previously sequenced isolate PHEV-Gent/PS-412/2020. The Bovine coronavirus reference strain (OP820535) was added to serve as an outgroup. The sequences were aligned using MAFFT (v7.490) (45). IQ-TREE (v2.3.2) was used to select the best-fit evolutionary model and to construct the maximum likelihood tree (46, 47). Branch support was assessed using ultrafast bootstrapping. The tree figure was annotated and visualized using iTOL (v6) (48).

**References:**

45) Katoh K, Standley DM. MAFFT Multiple Sequence Alignment Software Version 7: Improvements in Performance and Usability. Mol Biol Evol. 2013;30(4):772-780. doi:10.1093/molbev/mst010

46) Minh BQ, Schmidt HA, Chernomor O, et al. IQ-TREE 2: New Models and Efficient Methods for Phylogenetic Inference in the Genomic Era. Mol Biol Evol. 2020;37(5):1530-1534. doi:10.1093/molbev/msaa015

47) Kalyaanamoorthy S, Minh BQ, Wong TKF, von Haeseler A, Jermiin LS. ModelFinder: fast model selection for accurate phylogenetic estimates. Nat Methods. 2017;14(6):587-589. doi:10.1038/nmeth.4285

48) Cain B, Webb J, Yuan Z, et al. Prediction of cooperative homeodomain DNA binding sites from high-throughput-SELEX data. Nucleic Acids Res. 2023;51(12):6055-6072. doi:10.1093/nar/gkad318
